# Integrated Community‐Based Reporting and Field Diagnostics for Improved Rabies Surveillance in Rural Laikipia, Kenya

**DOI:** 10.1111/zph.13193

**Published:** 2024-12-02

**Authors:** Christian O. Odinga, Lian F. Thomas, Evalyne Wambugu, Adam W. Ferguson, Eric M. Fèvre, Andy Gibson, James M. Hassell, Dishon M. Muloi, Suzan Murray, Andrea Surmat, Peter M. Mwai, Rosie Woodroffe, Dedan Ngatia, Peter M. Gathura, John Waitumbi, Katherine E. L. Worsley‐Tonks

**Affiliations:** ^1^ International Livestock Research Institute Nairobi Kenya; ^2^ Smithsonian's National Zoo and Conservation Biology Institute Washington DC USA; ^3^ Department of Public Health, Pharmacology and Toxicology University of Nairobi Nairobi Kenya; ^4^ The Royal (Dick) School of Veterinary Studies University of Edinburgh Midlothian UK; ^5^ Institute of Infection, Veterinary and Ecological Sciences University of Liverpool Liverpool UK; ^6^ Kenya Medical Research Institute/US Army Medical Research Directorate–Africa/Kenya Kisumu Kenya; ^7^ Gantz Family Collection Center Field Museum of Natural History Chicago Illinois USA; ^8^ Mission Rabies Cranborne UK; ^9^ Department of Epidemiology of Microbial Disease Yale School of Public Health New Haven Connecticut USA; ^10^ Mpala Research Centre Nanyuki Kenya; ^11^ County Department of Veterinary Services Nanyuki Laikipia County Kenya; ^12^ Institute of Zoology Zoological Society of London London UK; ^13^ Department of Zoology & Physiology University of Wyoming Laramie Wyoming USA

**Keywords:** community‐based surveillance, field‐based diagnostic testing, one health, rabies, whole genome sequencing

## Abstract

Rabies vaccination in domestic dog populations has increased globally in a bid to protect human health. Surveillance efforts, however, are inconsistent in endemic regions such as in sub‐Saharan Africa, due to fragmented reporting and limited diagnostic capacity for suspected cases, limiting successful monitoring and evaluation of vaccination campaigns. Here, we conducted a pilot study aiming to strengthen rabies surveillance by combining community‐based surveillance with field‐based diagnostic testing in pastoral and agro‐pastoral communities in central Kenya; communities which are frequently marginalised from health systems. During the 6‐month pilot study, there were 14 alerts of suspected rabid dogs in the community, of which eight were tested and five diagnostically confirmed as rabid. Two positive samples processed successfully for whole genome sequencing indicated that the rabies variant circulating in central Kenya during the study period belonged to the Africa 1b subclade, which is similar to variants identified in eastern Kenya and Tanzania, suggesting regional transmission. This pilot study indicates that rabies continues to circulate in the region and that community‐based surveillance, when combined with enhanced diagnostic testing, can help alleviate underreporting and guide vaccination campaigns.

**Summary**

- The detection of several confirmed rabid dogs in the region demonstrates a need to strengthen dog vaccination and surveillance.
- Community‐based surveillance and rapid diagnostic tests can help reduce underreporting issues in remote and rural areas.
- The rabies virus variant identified in Laikipia is related to strains circulating in eastern Kenya indicating regional transmission.

## 1 Introduction

Rabies is a fatal zoonosis with an estimated annual human mortality of 59,000 globally (Hampson et al. 2015). In Africa, domestic dogs ( *Canis familiaris* ) are the main reservoirs of the rabies virus (RABV). To control rabies in dog populations and protect humans, mass dog vaccination is currently the most efficient strategy (Wallace et al. 2017), and a key pillar required to achieve the World Health Organisations' (WHO) target of eliminating dog‐mediated human rabies by 2030. This target has resulted in an increase in the number of African countries committing to rabies elimination by 2030 and in an increase in the number of dog vaccination campaigns in sub‐Saharan Africa (World Health Organization 2019). However, while there has been great progress on the vaccination front, rabies surveillance frequently lags behind (Taylor et al. 2017; Broban et al. 2018; Sambo et al. 2022). This is partly due to challenges in identifying human and dog rabies cases because of fragmented reporting in the community, and an inability to diagnostically confirm RABV. Yet improving RABV diagnostic capacity can enhance rabies surveillance (Taylor and Knopf 2015) as well as inform timely redistribution of post‐exposure prophylaxis (PEPs) for dog bite victims, especially in rural areas where there are fewer facilities that are able to store PEPs. Additionally, poor accountability of rabies cases hampers effective evaluation of vaccination campaigns (Franka and Wallace 2018). Weak surveillance may thus result in public health threats with fatal outcomes due to the misconception that rabies has been effectively controlled.

In Kenya, rabies continues to kill over 2000 people annually (Bitek et al. 2019), but this is likely an underestimate due to underreporting (Bitek et al. 2019). Indeed, while rabies surveillance in Kenya follows a passive surveillance structure, suspected rabid dogs are often killed by the community and dog bite victims often delay in seeking health care, leading to low levels of reporting across the country (Obonyo et al. 2016; Ngugi et al. 2018). Active community‐based surveillance and accompanying field‐based diagnostic testing including molecular characterisation of RABV would help reduce underreporting as well as guide vaccination campaigns.

In this study, we piloted an integrated community‐based surveillance system during a 6‐month period (June−November 2022) in Laikipia, Kenya, to explore the value of implementing a community‐integrated rabies surveillance approach. We focused our surveillance efforts on pastoral and agro‐pastoral communities because these communities are frequently overlooked in national disease surveillance systems (Doras et al. 2024; Hassell et al. 2020) despite evidence that rabies vaccination and surveillance must be augmented (Franka and Wallace 2018; Bitek et al. 2019).

## 2 Materials and Methods

### 2.1 Community‐Based Surveillance System

**FIGURE 1:**
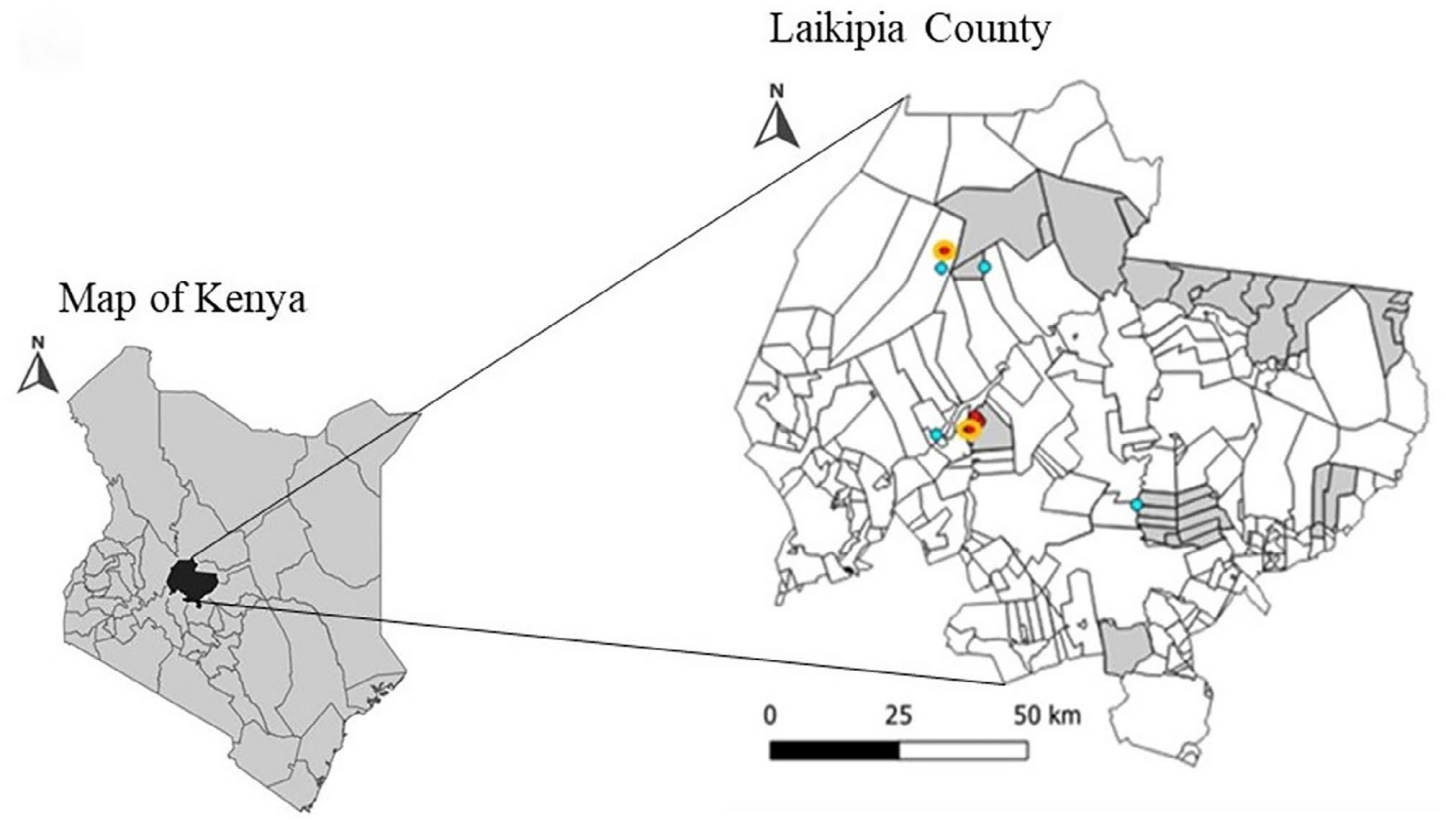
Map of Kenya and an inset showing location of Laikipia. Grey polygons indicate locations where active surveillance took place. Red dots show sampled dogs that were positive for rabies in the field test and the gold standard (red dot circled in yellow) while blue dots indicate locations of the sampled dogs that were negative for rabies.

**FIGURE 2:**
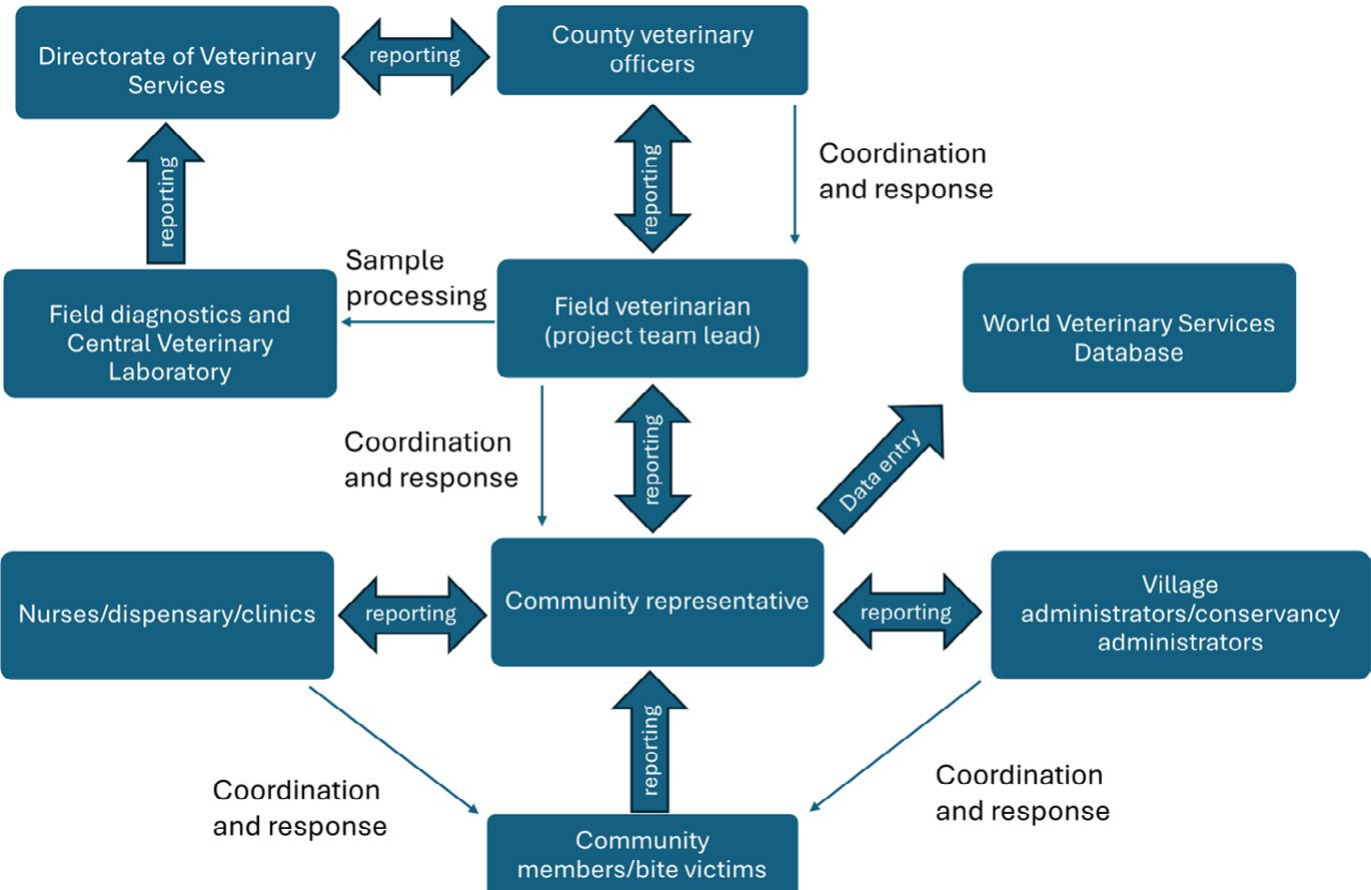
A schematic figure of the project surveillance structure. Note that this does not include the routine reporting that happens between the involved stakeholders.

Continuous‐integrated rabies surveillance was implemented in nine communities in Laikipia (Figure 1). Nine community representatives performed community‐based surveillance (one from each community) and were trained to identify and alert on dog bites, sick and dead suspected rabid dogs. The community representatives patrolled their respective regions three times a week for 6 months between June and November 2022. They met with different community members and nurses at local clinics/dispensaries to help identify cases of dog bites in humans, sick dogs, and dead dogs from road kills and unknown causes of death. Each case was reported in a database using the World Veterinary Services (WVS) application software (https://wvs.org.uk/) as well as notification to a field veterinarian from our team, through phone calls and sending photos or videos of sick or dead dogs. All community representatives involved in this pilot were paid, and mobile and transport costs associated with the work were covered. County veterinary services supported the surveillance and response when available (Figure 2). For each dog carcass, three portions of brain stem samples were collected: (1) for field‐based lateral flow immunochromatographic assay (LFA) testing; (2) for direct fluorescent antibody (DFA) testing; and (3) for virus whole genome sequencing.

### 2.2 Field‐Based Diagnostic Testing and Laboratory Confirmation of RABV

LFA is increasingly being proposed to strengthen rabies surveillance systems particularly in rural and remote areas that frequently suffer from underreporting due to cold‐chain limitations when shipping samples to large cities (Yale et al. 2019). In this study, we followed the protocol described in (Yale et al. 2019). For the DFA testing, samples were shipped to the Central Veterinary Laboratories in Nairobi, Kenya, where standard DFA protocols were used (Bitek et al. 2019).

### 2.3 Virus Molecular Characterisation

Whole genome sequencing was performed at the Kenya Medical Research Institute/ Walter Reed Project‐Kisumu, Kenya as described by Wambugu et al. (2024). Briefly, nucleic acids were processed to eliminate host DNA, followed by the synthesis and amplification of cDNA. The resulting dsDNA was purified and used for library preparation. Sequencing was performed on the NextSeq 2000 platform (Illumina, CA, USA). Raw sequence reads were processed and aligned to a reference RABV genome (KY210291). Samtools v0.1.19 (Li et al. 2009) was used to generate consensus genomes and visualise genome coverage. RABV‐GLUE tool (http://rabv‐glue.cvr.gla.ac.uk/) was used to assign the RABV to major and minor clades. The generated phylogenetic trees were visualised and annotated using Figtree v1.4.2 (Rambaut 2009). Raw sequence data generated in this study are available from the National Centre for Biotechnology Information (NCBI) Sequence Read Archive (SRA) under BioProject ID: OR359468 and OR359469.

## 3 Results

**FIGURE 3:**
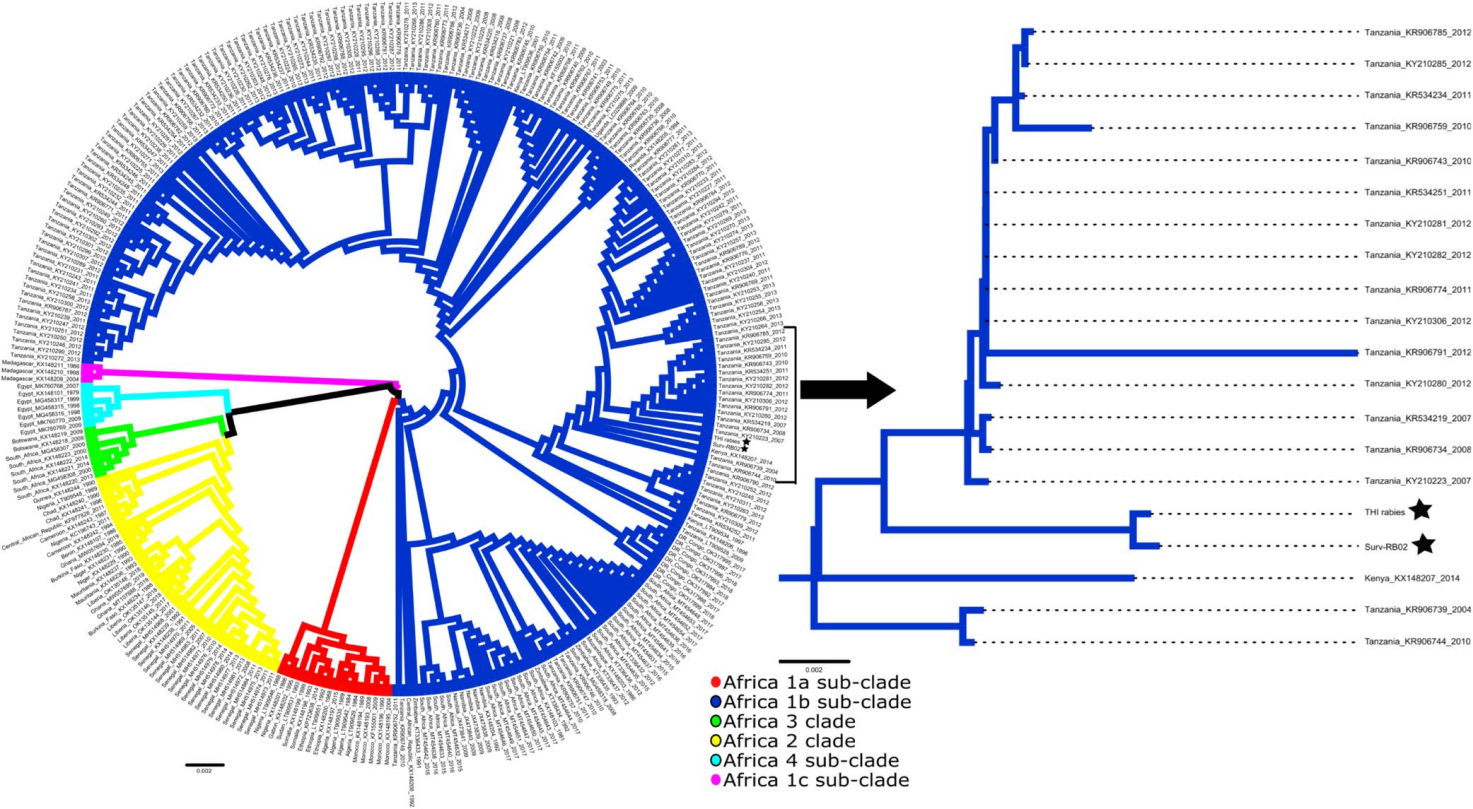
Maximum likelihood phylogeny of the major and minor clades of the two study genomes (shown as black stars) and other African RABV genomes available in the BV‐BRC database. The two study genomes clustered with the Cosmopolitan clade, in the Africa 1b subclade (shown in blue lines). The arrow shows a branch section of Africa 1b subclade that clustered with the two study genomes from the study area. Samples are colour coded by sub‐clade and clade. Red = Cosmopolitan Africa 1a sub‐clade, Blue = Cosmopolitan Africa 1b sub‐clade, Green = Africa 3 clade, yellow = Africa 2 clade, cyan = Cosmopolitan Africa 4 sub‐clade and Magenta = Cosmopolitan Africa 1c sub‐clade. The scale bar represents genetic distance.

Fourteen suspected rabid dogs were reported based on key signs that included unexplained aggression, biting, hyper‐salivation, unstable gait, hydrophobia, inappetence and sudden death. Brain samples were collected from eight of the 14 dead suspected rabid dogs, which were found dead not more than 12 h after reporting (which were either killed by community members or died from unknown causes). Testing was not done on the remaining six suspected cases because carcasses were either not found (two out of six) or were decomposed (four out of six). Results using LFA indicated that three (37.5%) of the eight suspected rabid dogs were positive for RABV while with DFA, five (62.5%) of the eight suspected rabid dogs were positive for RABV. LFA and DFA results are shown in Table S1. LFA had a sensitivity of 40% (11.8%–76.9%) and a specificity of 66.67% (20.8%–93.9%). The Cohen's Kappa for the agreement between the LFA and DFA tests is approximately 0.0587. All confirmed cases were reported from western Laikipia during the months of September and October 2022 (Figure 1). Whole genome sequencing was performed on two of the five samples that were successfully processed for genetic material. Two complete RABV genomes belonging to the Africa 1b subclade (Cosmopolitan clade) were identified (Figure 3).

## 4 Discussion

By piloting a small‐scale community‐based surveillance system, we detected several suspected rabies cases in domestic dogs some of which we diagnostically confirmed through field‐ and laboratory‐based techniques. The genetic profile of two samples was characterised, adding to the epidemiological understanding of rabies in Kenya.

The fact that more than 14 suspected rabid dogs were detected over the course of 6 months in a region of Laikipia where there have been very few reports of suspected rabid dogs in the last several years indicates that establishing continuous community‐based surveillance is warranted. The advantage of community‐based surveillance for zoonotic diseases in pastoral and agropastoral communities that are frequently marginalised from national health systems (Doras et al. 2024) has been highlighted in the past including for rabies (Bourhy et al. 2010), and our work adds evidence to this necessity. However, while WVS is a valuable data reporting and near‐real‐time data sharing platform, future work should have reporting that is integrated into existing national surveillance systems. Additionally, in this pilot, community representatives reported alerts via phone calls, and we recommend that future long‐term surveillance efforts establish alerts in the mobile application (with photos/videos included) that can be directly sent to local veterinarians and public health professionals. This will allow for faster One Health coordinated response and efficient use of resources for rabies surveillance and control. More broadly, for community‐based surveillance to effectively detect suspected rabid dogs in the community, there is an urgent necessity to better integrate community representatives into existing health systems, which includes compensation and regular communication with health authorities among other matters (Pallas et al. 2013; Kok et al. 2015). This will ensure the sustainability of these early‐warning surveillance efforts.

Findings from LFA results were in agreement with the gold‐standard DFA findings 50% of the time (sensitivity: 40% 11.8–76.9) and specificity: 66.67% (20.8–93.9) which is lower than what has been demonstrated elsewhere (Yale et al. 2019). Due to the small sample size (*n* = 8), it is challenging to identify the cause of these discrepancies and we recommend that the LFA approach be further evaluated with a larger sample size and in varying field conditions. From the molecular findings, the study shows close genetic relatedness between RABV strains in Africa 1b from Laikipia County, eastern Kenya (Wambugu et al. 2024), and those from Tanzania (Campbell et al. 2022), indicating a probable regional transmission of RABV strains. To the best of our knowledge, this is the first genetic characterisation of RABV in Laikipia, and while only two samples could be sequenced due to amplification challenges, these data will support rabies epidemiological investigations conducted at the national level.

In conclusion, the findings from this pilot study demonstrate the utility of implementing community‐based surveillance for augmenting suspected rabid dog case detections in rural and hard‐to‐reach pastoral and agropastoral communities, lending support for integrating such surveillance efforts into national rabies surveillance and control measures (Franka and Wallace 2018; Hassell et al. 2020; Worsley‐Tonks et al. 2022). As well as making a strong case for the need for continuous active surveillance, these findings highlight the need for concerted effort to reach appropriate vaccination coverages that are currently too low in this region (Ferguson et al. 2020). Finally, this work demonstrates the importance of combining active integrated community‐based surveillance and field‐based diagnostic testing into national surveillance systems, for raising the profile of neglected zoonoses in the under‐resourced rural settings of Africa.

## Ethics Statement

This work was approved by the International Livestock Research Institute's Institutional Animal Care and Use Committee (ILRI‐IACUC2022‐09), Laikipia's Directorate of Veterinary Services and Directorate of Public Health.

## Conflicts of Interest

The authors declare no conflicts of interest.

## Supporting information


Appendix S1.

## Data Availability

The data that support the findings of this study are available on request and will be provided in a public repository (GitHub) upon acceptance for publication.
