# Supplementary material for: Integrated Community‐Based Reporting and Field Diagnostics for Improved Rabies Surveillance in Rural Laikipia, Kenya
Source: Zoonoses Public Health. 2024 Dec 2;72(2):194–9. doi: 10.1111/zph.13193 (PMC11772914; doi:10.1111/zph.13193)
Supplement: Supplementary file 1 — Appendix S1. [file ZPH-72-194-s001.docx]

**Table S1: Description of sample diagnostic test results using the Lateral Flow Assay (LFA) and the Direct Fluorescent Antibody (DFA) tests.** An indication of which samples were submitted for sequencing is also provided.

| Sample ID | LFA result | DFA result | Molecular analysis |
| --- | --- | --- | --- |
| 1_23_9_22 | Positive | Positive | Yes |
| 2_17_9_22 | Positive | Positive | Yes |
| 3_10_3_22 | Negative | Positive | No |
| 4_10_3_22 | Negative | Positive | No |
| 6_10_11_22 | Negative | Positive | No |
| 7_20_10_22 | Positive | Negative | NA |
| 8_16_11_22 | Negative | Negative | NA |
| 8_17_11_22 | Negative | Negative | NA |
